# Automatic classification of long-term ambulatory ECG records according to type of ischemic heart disease

**DOI:** 10.1186/1475-925X-10-107

**Published:** 2011-12-14

**Authors:** Aleš Smrdel, Franc Jager

**Affiliations:** 1 University of Ljubljana, Faculty of Computer and Information Science, Tržaška 25, 1000 Ljubljana, Slovenia

## Abstract

**Background:**

Elevated transient ischemic ST segment episodes in the ambulatory electrocardiographic (AECG) records appear generally in patients with transmural ischemia (e. g. Prinzmetal's angina) while depressed ischemic episodes appear in patients with subendocardial ischemia (e. g. unstable or stable angina). Huge amount of AECG data necessitates automatic methods for analysis. We present an algorithm which determines type of transient ischemic episodes in the leads of records (elevations/depressions) and classifies AECG records according to type of ischemic heart disease (*Prinzmetal's angina*; *coronary artery diseases excluding patients with Prinzmetal's angina*; *other heart diseases*).

**Methods:**

The algorithm was developed using 24-hour AECG records of the Long Term ST Database (LTST DB). The algorithm robustly generates ST segment level function in each AECG lead of the records, and tracks time varying non-ischemic ST segment changes such as slow drifts and axis shifts to construct the ST segment reference function. The ST segment reference function is then subtracted from the ST segment level function to obtain the ST segment deviation function. Using the third statistical moment of the histogram of the ST segment deviation function, the algorithm determines deflections of leads according to type of ischemic episodes present (elevations, depressions), and then classifies records according to type of ischemic heart disease.

**Results:**

Using 74 records of the LTST DB (containing elevated or depressed ischemic episodes, mixed ischemic episodes, or no episodes), the algorithm correctly determined deflections of the majority of the leads of the records and correctly classified majority of the records with Prinzmetal's angina into the *Prinzmetal's angina *category (7 out of 8); majority of the records with other coronary artery diseases into the *coronary artery diseases excluding patients with Prinzmetal's angina *category (47 out of 55); and correctly classified one out of 11 records with other heart diseases into the *other heart diseases *category.

**Conclusions:**

The developed algorithm is suitable for processing long AECG data, efficient, and correctly classified the majority of records of the LTST DB according to type of transient ischemic heart disease.

## Background

Myocardial ischemia is an adverse outcome of pathologies, which compromise blood flow to the myocardium. It is a state when there is insufficient supply of oxygenated blood or the demand for it is too great. This can cause a part of the heart muscle to become electrically inactive, and can lead to acute myocardial infarction, and in worst case even death. On electrocardiogram (ECG) ischemia is manifested as transient change of ST segment level and morphology (transient ischemic ST segment episodes). In addition to these ischemic episodes, transient non-ischemic ST segment changes also appear [1,2]. These non-ischemic changes include: changes of ST segment level and morphology due to changes in heart rate (heart-rate related ST segment episodes); sudden changes of ST segment level due to sudden shifts of the electrical axis of the heart (axis shifts) or changes in ventricular conduction (conduction changes); and slow drifts of ST segment level due to diurnal changes or effects of medications. According to shift of ST segment level (positive or negative), transient ischemic ST segment episodes are either elevated or depressed.

Transient ischemic ST segment elevations typically appear in patients with acute transmural ischemia [1] and in patients where acute transmural ischemia without infarction occurs in the settings of Prinzmetal's angina [3,4]. Furthermore, persistent ST segment elevations indicate higher risk of mortality [5-10], a possible myocardial injury [11-13], and often (but not always) an acute myocardial infarction [14]. In some patients with ST segment elevations, reciprocal ST segment depressions may appear in leads that are separate and distinct from leads manifesting ST segment elevations [1,4]. These reciprocal changes can appear in leads reflecting contra lateral surface of the heart [1,4] or are believed to be secondary to coexisting distant ischemia, a manifestation of infarct extension, or may be an electrophysiological phenomenon caused by displacement of the injury current vector away from the non-infarcted myocardium [15]. In contrast to the transient ST segment elevations, the transient ST segment depressions appear in patients with other heart diseases, such as classic stable and unstable angina [3,4].

Monitoring of a patient over a prolonged time is needed in order to identify or observe sporadic transient events and to asses the extent and severeness of ischemic heart disease. The long-term ambulatory ECG (AECG) records thus obtained have to be checked and analyzed. Huge amount of data dictates use of automated methods for processing and evaluation of such records. As a diagnostic tool for a cardiologist it would be useful, time-saving and helpful to automatically determine "deflections of leads" of AECG records: *positive *(only elevated transient ischemic ST segment episodes present), *negative *(only depressed ischemic episodes), *mixed *(elevated and depressed ischemic episodes), or *no deflection *(no ischemic episodes); and then to automatically classify records according to "type of ischemic heart disease" into one of three categories: *Prinzmetal's angina *(*PMA*), *coronary artery diseases excluding patients with Prinzmetal's angina *(CAD*), and *other heart diseases *(*OHD*). This information could suggest further course of action such as additional investigations or might trigger an early treatment of a patient. The objective of this study was to develop an algorithm to automatically determine deflections of leads of AECG records according to type of transient ischemic ST segment episodes present, and then to automatically classify records according to type of ischemic heart disease into three categories (*PMA*, CAD*, and *OHD*).

## Methods

For this study we used the AECG records of the Long-Term ST Database (LTST DB) [16], which is intended to develop and to evaluate automated ischemia detectors and to study physiological mechanisms responsible for myocardial ischemia. The LTST DB contains 68 2- and 18 3-lead 24-hour AECG records (altogether 190 AECG leads) sampled at a constant rate of 250 samples per second per channel (Δ*T *= 4 ms), with amplitude resolution of 200 levels per 1 mV. The records of the LTST DB underwent a considerable preprocessing phase [16], which was essential for human expert annotators to be able to derive reliable reference annotations. Each lead of the records contains reference annotations for transient ischemic and transient non-ischemic heart-rate related ST segment episodes, reference annotations for axis shifts, and reference annotations that define time-varying ST segment reference level (non-ischemic path) along the leads of the records [16]. By subtracting time-varying ST segment reference level, *r_A_*(*i*, *j*), where *i *denotes the lead number and *j *denotes the heart beat number, from actual ST segment level, *s_A_*(*i*, *j*), the ST segment deviation level (or the ST segment deviation function), *d_A_*(*i*, *j*), was obtained for each lead of the records. All these functions are stored in the files of the LTST DB. Transient ischemic and transient non-ischemic heart-rate related ST segment episodes were then annotated in the ST segment deviation functions by human expert annotators of the LTST DB. Transient ST segment episodes were annotated according to three annotation criteria. These criteria state that the episode begins when the magnitude of the ST segment deviation function first exceeds 50 *μ*V. Next, the ST segment deviation function must reach a magnitude of at least *V_min _μ*V throughout the interval of at least *T_min _*s. The episode ends when the ST segment deviation function becomes lower than 50 *μ*V, provided that it does not exceed 50 *μ*V in the following 30 s. Values for *V_min _*and *T_min _*differ according to three annotation protocols and are: *V_min _*= 75 *μ*V and *T_min _*= 30 s for the protocol A; *V_min _*= 100 *μ*V and *T_min _*= 30 s for the protocol B; and *V_min _*= 100 *μ*V and *T_min _*= 60 s for the protocol C.

For this study we chose reference annotations according to annotation protocol B. For each lead of each record of the LTST DB, we verified the reference episode annotations at the extrema of each episode and determined deflections of leads. If the extrema of all ischemic episodes in a lead are positive, the deflection of lead is *positive*; if the extrema of all ischemic episodes in the lead are negative, the deflection of lead is *negative*; if ischemic episodes in the lead have negative as well as positive extrema, the deflection of lead is *mixed*; and if there are no ischemic episodes in the lead, the deflection of lead is *no episodes*. We considered all 86 records of the LTST DB. Of these, we used 74 records for the study, while we excluded those 12 records which contain non-ischemic heart-rate related ST segment episodes in each of their leads. Based on the knowledge of the expert cardiologists [1,3,4] we defined a set of rules by which AECG records can be classified into classes or categories of type of ischemic heart disease (*PMA *, CAD*, *OHD*) according to their deflections of leads. The set of rules are following:

(1) $$D_{p}=\left\{\begin{array}{c} PMA:\text{if}\;\exists i:O_{p}\left(i\right)=positive\\ {CAD}^{*}:\text{if}\;\exists i,\forall l,l\neq i:O_{p}\left(i\right)=negative\wedge \\ \;O_{p}\left(l\right)\in \left\{negative,\;mixed,no\;episodes\right\}\\ OHD:\text{if}\;\forall i:O_{p}\left(i\right)\in \text{\{}mixed,\;no\;episodes\text{\} ,} \end{array}\right)$$

where *D_p _*represents category into which a record *p *is classified, *O_p_*(.) represents determined deflection of a lead, while *i *and *l *represent lead numbers. A record containing at least one lead with *positive *deflection of lead is classified into the *PMA *category. A record containing at least one lead with *negative *and no leads with *positive *deflection of lead is classified into the CAD* category. And a record containing only leads with *mixed *or *no episodes *deflection of leads is classified into the *OHD *category. The 74 AECG records of the LTST DB used in this study, classified manually (based on the reference episode annotations of the database set by human expert annotators) into the categories of type of ischemic heart disease (*PMA*, CAD*, *OHD*), when using the set of rules (1) and the deflection of leads, are shown in Table 1. The records are also grouped according to the diagnoses.

**Table 1:** Manual classification of the records of the LTST DB

LTST DB (74 records)					
	
Type of heart disease ↓	Diagnoses				
	
	Prinzmetal's angina	Unstable angina	Angina	CAD	Other
*PMA*	6	1	1	0	0

CAD*	1	5	4	39	3

*OHD*	1	1	0	4	8

The 74 AECG records of the LTST DB with corresponding heart diseases classified manually according to defined rules into categories of type of ischemic heart disease. Records in column Other belong to patients with other diseases, either syndrome X, palpitations, hypercholesterolemia, syncope, hypertension, or cardiomyopathy. *Legend: *CAD - coronary artery disease.

The developed algorithm to classify AECG records into the categories of type of ischemic heart disease is composed from three modules: A. preprocessing; B. tracking of slow drifts, detection of axis shifts and correcting the ST segment reference level; and C. determining the deflection of leads and classifying the records according to type of ischemic heart disease.

### A. Preprocessing

The input to the developed algorithm were raw AECG data of the records and the ARISTOTLE's [17] fiducial points of normal and non-noisy heart beats which passed the preprocessing phase of the LTST DB [16]. These data are freely available to the users of the LTST DB. To further avoid the effects of noise, the average heart beats were constructed. Each normal heart beat in the raw AECG signal was replaced with the average heart beat. For the construction of each average heart beat normal non-noisy heart beats in the 16 s neighborhood of the current heart beat were used. Heart beats were aligned according to their fiducial points, *FP*(*i*, *j*), where *i *denotes lead number, and *j *denotes heart beat number. The *FP*(*i*, *j*) is located in QRS complex region of the *j*-th heart beat in the 'center of mass' of deflections [16].

To construct the ST segment level function, the algorithm searches for the positions of the isoelectric level and J point in each average heart beat. To determine the position of the isoelectric level of the *j*-th heart beat, *I*(*j*), the algorithm searches from the *FP*(*i*, *j*) backwards to point *FP*(*i*, *j*) - 108 ms in each lead for the "flattest" 20 ms segment of the signal and then determines one final position, *I*(*j*), [18,19]. For the position of the J point of the *j*-th heart beat, *J*(*j*), the algorithm searches forward from the *FP*(*i*, *j*) to the point *FP*(*i*, *j*) + 68 ms in each lead for a part of a waveform which "starts to flatten". One final position, *J*(*j*), is then determined as that furthest from the *FP*(*i*, *j*) [19,20].

**Figure 1:**
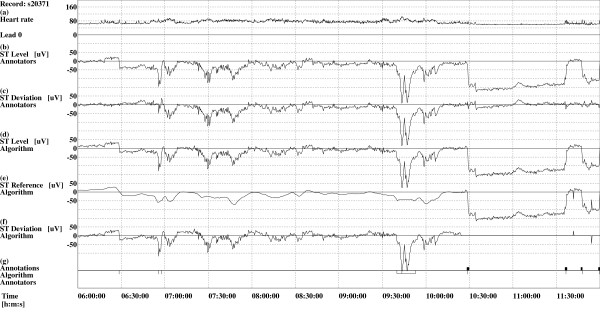
**Excerpt of a record s20371 from the LTST DB**. Time trends of the first lead of the 2-lead record s20371 from the LTST DB (6 hour excerpt from 24 hour record is shown, starting at 6 hours after the start of the recording), where deflection of lead was correctly determined as *negative*. *Legend*: (a) heart rate ([bpm]); (b) ST segment level function, *s*_A_(0, *j*), as derived by the human expert annotators of the LTST DB ([*μ*V]); (c) ST segment deviation function, *d*_A_(0, *j*), as derived by the human expert annotators ([*μ*V]); (d) ST segment level function, *s*(0, *k*), as derived by the algorithm ([*μ*V]); (e) ST segment reference function, *r*_2_(0, *k*), as derived by the algorithm ([*μ*V]); (f) ST segment deviation function, *d*(0, *k*), as derived by the algorithm ([*μ*V]); (g) Above the line: four axis shifts (vertical tics) as detected by the algorithm. Below the line: 7 axis shifts (vertical tics) and one transient ischemic ST segment episode (thin rectangle) as annotated by human expert annotators according to the protocol B of the database.

Using the positions of the isoelectric reference points and J points in the average heart beats, given lead, the algorithm constructs the ST segment level function, *s*(*i*, *j*), as:

(2) s(i,j)=a(i,j)-z(i,j),

where *a*(*i*, *j*) is the amplitude at the point of measurement of the ST segment level (*S*(*j*)), and *z*(*i*, *j*) is the amplitude of isoelectric level. Both amplitudes, *a*(*i*, *j*) and *z*(*i*, *j*), are determined as mean values of signal sample amplitudes in the 20 ms interval surrounding *S*(*j*) and *I*(*j*). The point of measurement of the ST segment level, *S*(*j*), is determined according to the position of the J point, *J*(*j*), and heart rate by following rule:

(3) $$S\left(j\right)=\left\{\begin{array}{c} J\left(j\right)+80\text{ms: if}\;\;\;\;\;HR\left(j\right)<100\text{ bpm}\\ J\left(j\right)+72\text{ms: if }100\text{ bpm}\leq HR\left(j\right)<110\text{ bpm}\\ J\left(j\right)+64\text{ms: if }110\text{ bpm}\leq HR\left(j\right)<120\text{ bpm}\\ J\left(j\right)+60\text{ms: if }120\text{ bpm}\leq HR\left(j\right), \end{array}\right)$$

where *HR*(*j*) denotes heart rate at the *j*-th heart beat, measured in beats per minute [bpm]. The ST segment level function, *s*(*i*, *j*), is then resampled at a constant rate of 0.5 samples per second and smoothed using 7-point moving average filter to obtain the "raw" ST segment level function, *s*(*i*, *k*), where *i *denotes the lead number and *k *denotes the sample number in the resampled function [19]. An example of the ST segment level function derived by the human expert annotators of the LTST DB and that derived by the algorithm is shown in Figure 1b and 1d respectively.

### B. Tracking of slow drifts, detection of axis shifts and correcting the ST segment reference level

In order to accurately determine deflection of leads, all non-ischemic events have to be excluded from further analysis. For this, the algorithm tracks the time-varying non-ischemic path in each ST segment level function along the record to create the ST segment reference function. The ST segment reference function is then subtracted from the ST segment level function to obtain the ST segment deviation function, which is used to determine deflection of leads. Construction of the ST segment reference function is made in several steps.

In the first step, the algorithm tracks slowly varying ST segment reference level trend by applying two moving average filters of 6 h 40 min (*h_g_*) and 5 min (*h_l_*) in length to the ST segment level function, *s*(*i*, *k*), to obtain the time-varying global, *r_g_*(*i*, *k*), and local, *r_l_*(*i*, *k*), ST segment reference level trends, respectively. The lengths of the impulse responses of the filters were selected such that the output of *h_g _*models slow changes of the ST segment level function (e. g. slow drifts), while output of *h_l _*models faster changes of the ST segment level function (e. g. transient ST segment episodes) [21]. Using these two ST segment reference level trends, the algorithm obtains the estimation of the ST segment reference function, *r*_1_(*i*, *k*), which tracks slow drifts but skips faster events and episodes. The *r*_1_(*i*, *k*) is taken as *r_g_*(*i*, *k*) if the absolute difference between the *r_g_*(*i*, *k*) and *r_l_*(*i*, *k*) is more than 50 *μ*V; otherwise it is taken as *r_l_*(*i*, *k*).

In the second step, axis shifts due to position changes and changes in ventricular conduction are detected in each ECG lead as a step change in the ST segment level function and Mahalanobis distance functions of the first order of the QRS complex and of the ST segment Karhunen-Loève coefficients feature vectors. These distance functions are also included in the LTST DB and are available to the users of the database. Axis shifts are detected as a step change within *T_a _*= 72 s interval which has a flat interval of length *T_f _*= 216 s before and after the step change. The final ST segment reference function, *r*_2_(*i*, *k*), is obtained by replacing the *r*_1_(*i*, *k*) with *s*(*i*, *k*) in the intervals surrounding the detected axis shifts, forward and backward from the axis shifts, until the absolute difference between the *r_g_*(*i*, *k*) (or *r_l_*(*i*, *k*)) and *s*(*i*, *k*) is less then 50 *μ*V [21]. By subtracting the ST segment reference function of the lead from the ST segment level function we get the ST segment deviation function, where slow drifts and axis shifts due to body position changes and changes in ventricular conduction are excluded:

(4) $$d\left(i,k\right)=s\left(i,k\right)-r_{2}\left(i,k\right).$$

Ideally, we would get the ST segment deviation function where only transient ST segment episodes are present. The obtained ST segment reference function of the example from Figure 1 (Figure 1e) tracks the non-ischemic changes quite well, so the ST segment deviation function derived by the algorithm (Figure 1f) is quite similar to that constructed by the human expert annotators of the LTST DB (Figure 1c).

### C. Determining the deflection of leads and classifying the records according to type of ischemic heart disease

**Figure 2:**
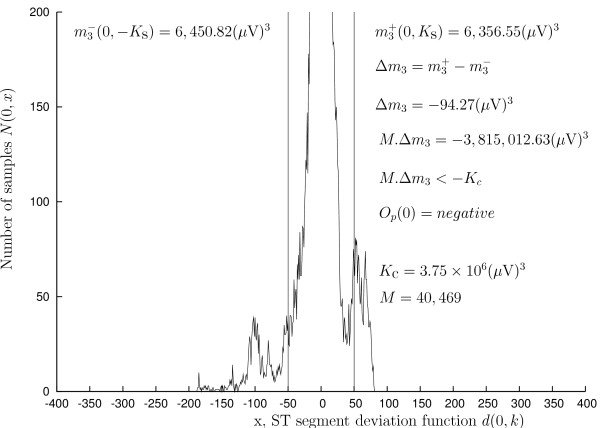
**Example histogram of the ST segment deviation function**. Histogram of the ST segment deviation function of the first lead, *d*(0, *k*), of the record s20371 of the LTST DB. Transient ischemic ST segment episodes in this lead are depressions. See also Figure 1 and text.

To determine the deflection of leads, the samples of the ST segment deviation function of a lead, *d*(*i*, *k*), are considered as samples of a random variable, and are used to construct a histogram of this function. An example demonstrating histogram of the first lead of the record s20371 of the LTST DB is shown in Figure 2. Then, the *z*-th statistical moment above the threshold *K_S _*= 50 *μ*V:

(5) $$m_{z}^{+}\left(i,K_{S}\right)=\overset{B}{\underset{x=K_{S}}{\sum }}\left(|x-K_{S}{|}^{z}.\frac{1}{M}.N\left(i,x\right)\right),$$

and *z*-th statistical moment below the threshold -*K_S_*:

(6) $$m_{z}^{-}\left(i,-K_{S}\right)=\overset{-K_{S}}{\underset{x=-B}{\sum }}\left(|x+K_{S}{|}^{z}.\frac{1}{M}.N\left(i,x\right)\right),$$

are computed, where *z *represents the statistical moment used (*z *= 1, 2, 3), *N*(*i*, *x*) the number of samples with the value *x *in the histogram, *M *the number of samples of *d*(*i*, *k*), and *B *= 1500 *μ*V defines the upper and lower bounds between which the histogram is constructed [21]. The algorithm determines the deflection of lead, *O_p_*(*i*), of the lead *i *of a record *p*, using the *z*-th statistical moments above and below the threshold *K_S_*, by applying the following rule [21]:

(7) $$O_{p}\left(i\right)=\left\{\begin{array}{c} positive:\\ \;\text{if (}m_{z}^{+}\text{(}i,K_{S}\text{)}-m_{z}^{-}\text{(}i,-K_{S}\text{))}>\frac{1}{M}.K_{C}\\ negative:\\ \;\text{if (}m_{z}^{+}\text{(}i,K_{S}\text{)}-m_{z}^{-}\text{(}i,-K_{S}\text{))}<-\frac{1}{M}.K_{C}\\ mixed\;or\;no\;episodes:\\ \;\text{otherwise,} \end{array}\right)$$

where *K_C _*is the threshold for lead classification and differs according to the statistical moment used. The threshold *K_c _*is the same for all leads of all records of the database, given statistical moment used for lead classification (either the first, or the second, or the third). The example in Figure 2 demonstrates determining deflection of the first lead of the record s20371 using the third statistical moment.

To optimize the algorithm, we investigated the first, second, and third statistical moment, for various values of threshold *K_C_*. The *K_C _*determines whether deflection of a lead is decided as *positive*, or *negative*, or as *mixed *or *no episodes*. Using higher *K_C _*more leads have deflections of leads determined as *mixed *or *no episodes*, while using lower *K_C _*more leads have deflections of leads determined as *positive *or *negative*.

As the main optimization constraint we took the minimum number of leads containing only elevated ischemic episodes being falsely determined as having *negative*, and the minimum number of leads containing only depressed ischemic episodes being falsely determined as having *positive *deflections of leads. We also tried to maximize the number of leads containing both types of ischemic episodes and leads without any ST segment episodes determined as having *mixed *or *no episodes *deflections of leads. We tested different values of the threshold *K_C _*for the first, second, and third statistical moment. The optimal values obtained for the threshold *K_C _*were: 2 × 10^3^(*μ*V) for the first, 75 × 10^3^(*μ*V)^2 ^for the second, and 3.75 × 10^6^(*μ*V)^3 ^for the third statistical moment.

Finally, the algorithm automatically classifies each record *p *into one of the categories of type of ischemic heart disease, *D_p _*∈ {*PMA*, CAD*, *OHD*}, using the set of rules (1).

## Results

Results of determining deflections of leads of the 74 AECG records of the LTST DB using different moments and optimal thresholds are shown in Table 2. The results when the reference level correction was applied are shown left, while when no reference level correction was applied are shown right. The upper left part shows the results of determining deflections of leads using the first statistical moment. The algorithm correctly determined deflections of 90% of leads (9 out of 10) with elevations as *positive*. The algorithm correctly determined deflections of 96% of leads (89 out of 93) with depressions as *negative*. For the leads with mixed types of episodes the algorithm determined deflections of 60% of leads (three out of five) as *positive *and of 40% of leads (two out of five) as *negative*. The algorithm also correctly determined deflections of 19% of leads without transient ST segment episodes (8 out of 43) as *mixed *or *no episodes*. The middle left part of Table 2 shows the results of determining deflections of leads using the second statistical moment. The algorithm correctly determined deflections of 100% of leads with elevations as *positive*, and of 97% of leads with depressions as *negative*. For the leads with mixed types of episodes the algorithm determined deflections of 60% of leads as *positive *and of 40% as *negative*. The algorithm also correctly determined deflections of 30% of leads without transient ST segment episodes as *mixed *or *no episodes*.

**Table 2:** Results of determining deflections of leads

	Reference level correction			No reference level correction		
**First moment**	***K_C _*= 2 × 10^3^(*μ*V)**			***K_C _*= 3.5 × 10^3^(*μ*V)**		
	
	***positive***	***negative***	***mixed, no episodes***	***positive***	***negative***	***mixed, no episodes***

Elevations	9 (90%)	0 (0%)	1 (10%)	8 (80%)	2 (20%)	0 (0%)

Depressions	3 (3%)	89 (96%)	1 (1%)	24 (26%)	67 (72%)	2 (2%)

Mixed	3 (60%)	2 (40%)	0 (0%)	2 (40%)	3 (60%)	0 (0%)

No episodes	11 (26%)	24 (56%)	8 (19%)	11 (26%)	27 (63%)	5 (12%)

**Second moment**	***K_C _*= 75 × 10^3^(*μ*V)^2^**			***K_C _*= 150 × 10^3^(*μ*V)^2^**		
	
	***positive***	***negative***	***mixed, no episodes***	***positive***	***negative***	***mixed, no episodes***

Elevations	10 (100%)	0 (0%)	0 (0%)	9 (90%)	1 (10%)	0 (0%)

Depressions	0 (0%)	90 (97%)	3 (3%)	17 (18%)	76 (82%)	0 (0%)

Mixed	3 (60%)	2 (40%)	0 (0%)	2 (40%)	3 (60%)	0 (0%)

No episodes	10 (23%)	20 (47%)	13 (30%)	12 (28%)	24 (56%)	7 (16%)

**Third moment**	***K_C _*= 3.75 × 10^6^(*μ*V)^3^**			***K_C _*= 4.75 × 10^6^(*μ*V)^3^**		
	
	***positive***	***negative***	***mixed, no episodes***	***Positive***	***negative***	***mixed, no episodes***

Elevations	10 (100%)	0 (0%)	0 (0%)	9 (90%)	1 (10%)	0 (0%)

Depressions	0 (0%)	92 (99%)	1 (1%)	12 (13%)	81 (87%)	0 (0%)

Mixed	3 (60%)	2 (40%)	0 (0%)	2 (40%)	3 (60%)	0 (0%)

No episodes	9 (21%)	17 (40%)	17 (40%)	13 (30%)	24 (56%)	6 (14%)

Results of determining deflections of leads of the 74 AECG records using the first, second, and third statistical moment, with the reference level correction (left) and without the reference level correction (right), with regard to the reference annotations of the protocol B of the LTST DB. Percentages of leads per total number of leads for given deflection (elevations, depressions, mixed, or no episodes) are bracketed.

The lower left part of Table 2 shows the results of determining deflections of leads using the third statistical moment. The algorithm correctly determined deflections of 100% of leads with elevations as *positive*, and of 99% of leads with depression as *negative*. For the leads with mixed types of episodes the algorithm determined deflections of 60% of leads as *positive *and of 40% as *negative*. The algorithm also correctly determined deflections of 40% of leads without transient ST segment episodes as *mixed *or *no episodes*. The right part of Table 2 shows results of determining deflections of leads using different statistical moments when no reference level correction was performed. Values of the threshold *K_C _*were optimized for best performance. Using the first statistical moment (upper right, refer also to left part of the Table 2), the algorithm correctly determined deflections of 80% of leads with elevations as *positive *and of 72% of leads with depressions as *negative*. The algorithm also correctly determined deflections of 12% of leads without transient ST segment episodes as *mixed *or *no episodes*. Using the second statistical moment (middle right), the algorithm correctly determined deflections of 90% of leads with elevations as *positive *and of 82% of leads with depressions as *negative*. The algorithm also correctly determined deflections of 16% of leads without transient ST segment episodes as *mixed *or *no episodes*. Finally, using the third statistical moment (lower right), the algorithm correctly determined deflections of 90% of leads with elevations as *positive *and of 87% of leads with depressions as *negative*. The algorithm also correctly determined deflections of 14% of leads without transient ST segment episodes as *mixed *or *no episodes*.

The best results were obtained using the third statistical moment which represents skewness of the distribution. The difference of the positive and negative moments above 50 *μ*V and below -50 *μ*V respectively shows which side lobe of distribution prevails e. g. whether there are significant elevations or depressions present. The algorithm differentiates very well between the leads containing only elevated or only depressed ischemic episodes, but is not yet suitable for use with leads containing mixed type of episodes. Next, using the third statistical moment for determining deflections of leads, the algorithm then classified the records using the set of rules (1) according to type of ischemic heart disease. These results are shown in Table 3. The table shows the results of manual classification of the records (upper), of automatic classification by the algorithm (middle), and the difference between the results of automatic and of manual classification (lower). The results are also grouped according to the diagnoses.

**Table 3:** Manual and automatic classification (with differences) of the records of the LTST DB

Reference: LTST DB (74 records)						
	
Type of heart disease ↓	Diagnoses					
	
	Prinzmetal's angina	Unstable angina	Angina	CAD		Other
*PMA*	6	1	1	0		0

CAD*	1	5	4	39		3

*OHD*	1	1	0	4		8

	Σ8	Σ55				Σ11
	
**Algorithm: LTST DB (74 records)**						
	
**Type of heart disease** **↓**	**Diagnoses**					
	
	**Prinzmetal's angina**	**Unstable angina**	**Angina**	**CAD**		**Other**

*PMA*	[7] Σ7	[2	2	2]	Σ6	6

CAD*	1	[5	3	39]	Σ47	4

*OHD*	0	[0	0	2]	Σ2	[1] Σ2

	Σ8	Σ 55				Σ11
	
**Algorithm - Reference**						
	
**Type of heart disease** **↓**	**Diagnoses**					
	
	**Prinzmetal's angina**	**Unstable angina**	**Angina**	**CAD**		**Other**

*PMA*	1	1	1	2		6

CAD*	0	0	-1	0		1

*OHD*	-1	-1	0	-2		-7

The 74 AECG records of the LTST DB with corresponding diagnosis, classified according to defined categories of type of ischemic heart disease. Results of manual classification are shown in the upper part, results of automatic classification in the middle, and the differences between automatic and manual classification in the lower part of the table. *Legend: *CAD - coronary artery disease.

The algorithm (middle) correctly classified 7 out of 8 patients with Prinzmetal's angina into *PMA *category, and one was misclassified into CAD* category. The algorithm classified 47 out of 55 patients with unstable angina, angina, or coronary artery disease into CAD* category, while 6 were misclassified into *PMA *and two into *OHD *category. One patient out of 11 with other heart disease was classified into *OHD *category. The difference between automatic and manual classification (bottom) shows, that the automatic classification gives pretty much similar results as the manual classification. Using the set of rules (1) the algorithm managed to correctly recognize majority of records belonging to patients with Prinzmetal's angina and majority of records belonging to patients with other coronary artery diseases.

## Discussion and Conclusions

**Figure 3:**
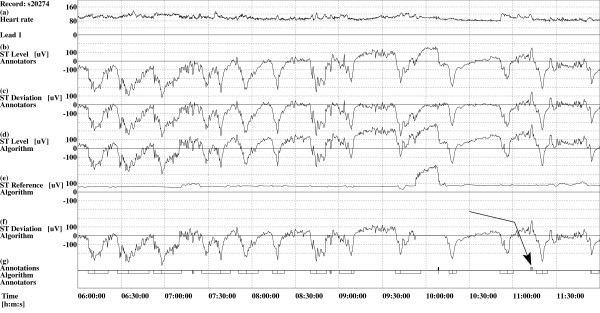
**Excerpt of a record s20274 from the LTST DB**. Time trends of the second lead of the 2-lead record s20274 from the LTST DB (6 hour excerpt from 24 hour record is shown, starting at 6 hours after the start of the recording), where deflection of lead was determined as *negative *instead of *mixed *or *no episodes*. *Legend*: (a) heart rate ([bpm]); (b) ST segment level function, *s*_A_(1, *j*), as derived by the human expert annotators of the LTST DB ([*μ*V]); (c) ST segment deviation function, *d*_A_(1, *j*), as derived by the human expert annotators ([*μ*V]); (d) ST segment level function, *s*(1, *k*), as derived by the algorithm ([*μ*V]); (e) ST segment reference function, *r*_2_(1, *k*), as derived by the algorithm ([*μ*V]); (f) ST segment deviation function, *d*(1, *k*), as derived by the algorithm ([*μ*V]); (g) Above the line: one axis shift (vertical tic, time 10 h 8 min) as detected by the algorithm and only one elevated transient ischemic ST segment episode (thin rectangle) as annotated by the human expert annotators (see the arrow). Below the line: one axis shift (vertical tic, time 10 h 8 min) and 15 depressed ischemic episodes (thin rectangles) as annotated by the human expert annotators according to the protocol B of the database.

The results show, that the developed algorithm can be used to classify patients according to type of ischemic heart disease. Using the set of rules (1) for classification of records the results of automatic classification were similar to those of manual classification, with the exception of classification of records in the "Other" category. Main reason for this is that some of those records contain non-ischemic events, which are not all properly detected and thus cause wrong classification. To rectify this we would need to improve the part of the algorithm responsible for detection and exclusion of non-ischemic events. The results showed that the proposed algorithm is efficient in determining deflections of leads with only elevated or depressed transient ischemic ST segment episodes present, but is not yet suitable for use with leads containing mixed type of episodes. Using the third statistical moment, the deflections of almost all leads with either elevated or depressed ischemic episodes were correctly determined. The algorithm did not perform well in determining deflections of leads in those leads which contain both types of transient ischemic ST segment episodes. This is mostly due to the fact that in these leads there is a large number of depressions and small number of elevations, or vice versa. Larger number of episodes of one type prevails and deflection of lead is then determined as either *positive *or *negative*. An example is the record s20274 from the LTST DB (see Figure 3). Both leads of this record have elevated as well as depressed ischemic episodes, but the algorithm determined deflections of both leads as *negative*. The first lead of this record contains three elevated and 36 depressed, while the second lead contains only one elevated and 62 depressed ischemic episodes. A 6-hour excerpt of the second lead of this record, with 15 depressed and one elevated ischemic episode is shown. The results of determining deflections of leads with no transient ST segment episodes were not good. The deflections of approximately one third of these leads (using the third statistical moment) were correctly determined as *mixed *or *no episodes*. Lower performance is due to the fact that some axis shifts were not detected, consequently causing wrong determination of deflections of leads.

Our algorithm performed exceptionally well in determining deflections of leads and in classifying patients according to type of ischemic heart disease, but there are still some limitations, which concern leads containing both types of transient ischemic ST segment episodes and leads without transient ST segment episodes. In cases where leads contain larger number of one type of ischemic episodes, the insertion of such leads into *mixed *group seems to be inadequate. A division of these records into more groups, or some other method for determining deflections of leads, might be considered, and the rule (7) for determining deflections of leads would need to be improved. The problem concerning leads without transient ST segment episodes is the inability of the algorithm to accurately detect all axis shifts. To rectify this, the developed method for detecting axis shifts would need to be improved. Other techniques for detecting axis shifts due to body position changes were investigated in the past [22-24]. Pitfalls with these techniques lie in the fact that they were developed using artificially triggered axis shifts, so prior validation of these techniques using real clinical data should be performed.

The algorithm shows high sensitivity of determining deflection of leads (100% for leads containing elevations only and 99% for leads containing depressions only) with some false positives. The proposed algorithm is efficient and could be a valuable aid in every day clinical practice. The algorithm is, despite some limitations, appropriate for processing large amount of AECG data and for quick assessment of type of ischemic heart disease. The study showed that the reference level correction (tracking of slow drifts, detection of axis shifts, and correcting the ST segment reference level) is an essential part of the algorithm and enables good classification of patients according to type of ischemic heart disease. Without the module for reference level correction, the deflections of a substantial part of leads were incorrectly determined. Early and accurate assessment of the deflection of leads itself is already valuable for a clinician, since this information suggests the cause and type of ischemia.

## Competing interests

The authors declare that they have no competing interests.

## Authors' contributions

Both authors have collaborated in this study. Together they designed and carried out the study. They also collaborated in drafting the manuscript. Both authors also read and approved the final manuscript.
